# Crystal structure of (2,4-di-*tert*-butyl-6-{[(6,6′-dimethyl-2′-oxido-1,1′-biphenyl-2-yl)imino]methyl}phenolato-κ^3^ *O*,*N*,*O*′)bis(propan-2-olato-κ*O*)titanium(IV)

**DOI:** 10.1107/S1600536814018455

**Published:** 2014-08-16

**Authors:** Liang Chen, Huiran Wang, Xuebin Deng

**Affiliations:** a Department of Chemistry, Beijing Normal University, Beijing 100875, People’s Republic of China; b Shanghai Tianshan High School, Shanghai 200336, People’s Republic of China

**Keywords:** crystal structure, titanium(IV) complex, 2-amino-2′-hy­droxy-6,6′-dimethyl-1,1′-biphen­yl, Schiff base ligand

## Abstract

In the mononuclear Ti^IV^ title complex, [Ti(C_29_H_33_NO_2_)(C_3_H_6_O)_2_], the TiNO_4_ coordination polyhedron comprises an N-atom and two O-atom donors from the dianionic Schiff base ligand and two O-atom donors from monodentate isopropoxide anions. The stereochemistry is distorted trigonal–bipyramidal with the N-donor in an elongated axial site [Ti—N = 2.2540 (17) Å], the O-donors having normal Ti—O bond lengths [1.7937 (14) Å (axial)–1.8690 (14) Å]. In the crystal, C—H⋯π inter­actions link mol­ecules into centrosymmetric dimers.

## Related literature   

For background information, see: Zi (2011 ▶). For a similar structure, see: Chen *et al.* (2013 ▶).
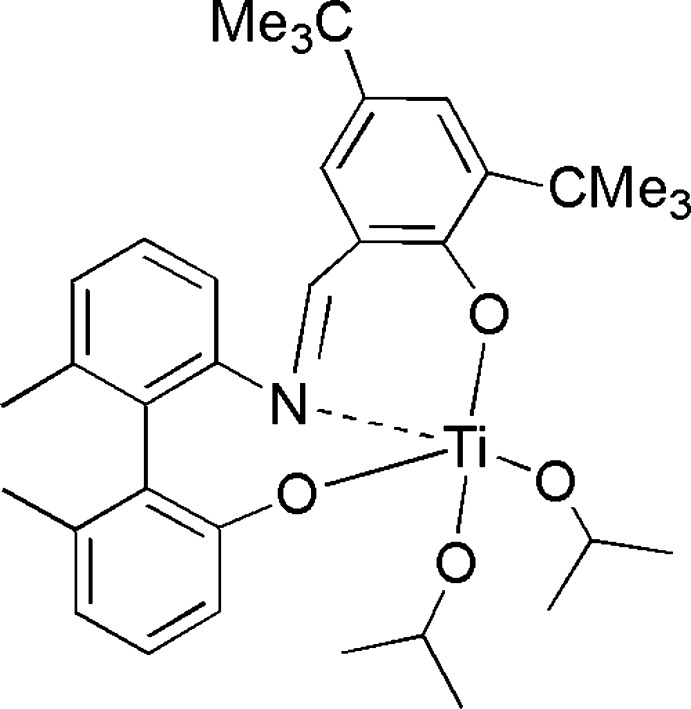


## Experimental   

### Crystal data  

[Ti(C_29_H_33_NO_2_)(C_3_H_6_O)_2_]
*M* *_r_* = 593.64
Triclinic, 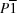

*a* = 11.5511 (11) Å
*b* = 11.9083 (10) Å
*c* = 12.6336 (11) Å
α = 80.941 (2)°
β = 72.869 (1)°
γ = 87.253 (2)°
*V* = 1640.0 (3) Å^3^
*Z* = 2
Mo *K*α radiation
μ = 0.30 mm^−1^
*T* = 110 K
0.50 × 0.49 × 0.21 mm

### Data collection  

Bruker CCD area-detector diffractometer
Absorption correction: multi-scan (*SADABS*; Bruker, 2009 ▶) *T*_min_ = 0.865, *T*_max_ = 0.940
8299 measured reflections
5896 independent reflections
4914 reflections with *I* > 2σ(*I*)
*R*_int_ = 0.022

### Refinement  

*R*[*F* ^2^ > 2σ(*F* ^2^)] = 0.043
*wR*(*F* ^2^) = 0.114
*S* = 1.05
5896 reflections
382 parameters
H-atom parameters constrained
Δρ_max_ = 0.31 e Å^−3^
Δρ_min_ = −0.36 e Å^−3^

### 

Data collection: *APEX2* (Bruker, 2009 ▶); cell refinement: *SAINT* (Bruker, 2009 ▶); data reduction: *SAINT*; program(s) used to solve structure: *SHELXS97* (Sheldrick, 2008 ▶); program(s) used to refine structure: *SHELXL97* (Sheldrick, 2008 ▶); molecular graphics: *SHELXTL* (Sheldrick, 2008 ▶); software used to prepare material for publication: *publCIF* (Westrip, 2010 ▶).

## Supplementary Material

Crystal structure: contains datablock(s) I, New_Global_Publ_Block. DOI: 10.1107/S1600536814018455/zs2308sup1.cif

Structure factors: contains datablock(s) I. DOI: 10.1107/S1600536814018455/zs2308Isup2.hkl

**Fig. 1.**
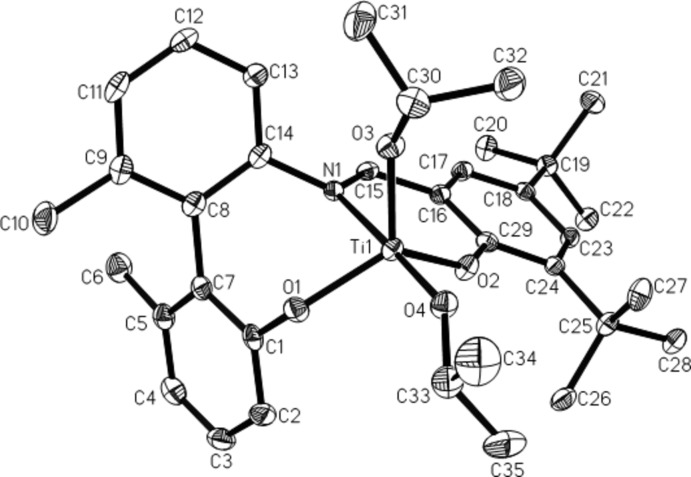
Molecular configuration and atom numbering scheme for the title complex, with displacenemt ellipsoids drawn at the 50% probability level. Hydrogen atoms omitted for clarity.

. DOI: 10.1107/S1600536814018455/zs2308fig1.tif
Mol­ecular configuration and atom numbering scheme for the title complex, with displacenemt ellipsoids drawn at the 50% probability level. Hydrogen atoms omitted for clarity.

CCDC reference: 922371

Additional supporting information:  crystallographic information; 3D view; checkCIF report

## Figures and Tables

**Table 1:** Hydrogen-bond geometry (Å, °) *Cg*1 is the centroid of the C16–C18, C23, C24, C29 ring.

*D*—H⋯*A*	*D*—H	H⋯*A*	*D*⋯*A*	*D*—H⋯*A*
C20—H20*A*⋯*Cg*1^i^	0.98	2.74	3.659 (3)	156

Symmetry code: (i) 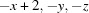.

## supplementary crystallographic information

### S1. Chemical context

Titanium complexes containing Schiff-base ligands are important catalysts with many applications such as the hydro­amination of alkenes (Zi, 2011). Titanium complexes derived from chiral 2-amino-2'-hy­droxy-6,6'-di­methyl-1,1'-bi­phenyl have been extensively studied (Zi, 2011; Chen *et al.*, 2013). The aim of our ongoing research on titanium complexes is to synthesize the titanium complexes with tridentate Schiff-base ligand. Herein, the synthesis and crystal structure of the title mononuclear Ti^IV^ complex with 2-(3,5-di-*tert*-butyl-2-oxyl­benzyl­idene­amino)-2'-oxy-6,6'-δi­methyl-1,1'-bi­phenyl and iso-propoxide, C_35_H_47_NO_4_Ti is reported.

### S2. Structural commentary

In the structure of the title complex (Fig. 1) the TiNO_4_ coordination polyhedron comprises a nitro­gen and two oxygen donors from the dianionic Schiff base and two O-atom donors from monodentate isopropoxide anions. The stereochemistry is distorted trigonal-bipyramidal with the N-donor in an elongated axial site [Ti—N = 2.2540 (17) Å], the O-donors with normal Ti—O bond lengths [1.7937 (14) Å (axial)–1.8690 (14) Å]. In the crystal, no inter­molecular inter­actions are found. For a related structure, see: Chen *et al.* (2013).

### S3. Synthesis and crystallization

A toluene solution (10 mL) of the Schiff base ligand *racemic*-2-(3,5-di-*tert*-butyl-2-hydroxyl­benzyl­idene­amino)-2'-hy­droxy-6,6'-di­methyl-1,1'-bi­phenyl (0.43 g, 1.0 mmol) was slowly added to a toluene solution (10 mL) of Ti(O*Pr*)_4_ (0.28 g, 1.0 mmol) with stirring at room temperature for one day, after which the solution was filtered. The filtrate was concentrated to about 2 mL under vacuum. Yellow crystals of the title complex were isolated from this solution after three days standing at room temperature. Yield: 0.44 g (74%).

### S4. Refinement details

The hydrogen atoms were positioned geometrically and refined using a riding model with *U*_iso_(H) = 1.2*U*_eq_(C) (aromatic) and 1.5 times *U*_eq_(C) (methyl).

### Crystal data

**Table:**

[Ti(C_29_H_33_NO_2_)(C_3_H_6_O)_2_]	*Z* = 2
*M**_r_* = 593.64	*F*(000) = 636
Triclinic, *P*1	*D*_x_ = 1.202 Mg m^−^^3^
*a* = 11.5511 (11) Å	Mo *K*α radiation, λ = 0.71073 Å
*b* = 11.9083 (10) Å	Cell parameters from 2631 reflections
*c* = 12.6336 (11) Å	θ = 2.5–27.5°
α = 80.941 (2)°	µ = 0.30 mm^−^^1^
β = 72.869 (1)°	*T* = 110 K
γ = 87.253 (2)°	Block, yellow
*V* = 1640.0 (3) Å^3^	0.50 × 0.49 × 0.21 mm

### Data collection

**Table:**

Bruker CCD area-detector diffractometer	5896 independent reflections
Radiation source: fine-focus sealed tube	4914 reflections with *I* > 2σ(*I*)
Graphite monochromator	*R*_int_ = 0.022
φ and ω scans	θ_max_ = 25.3°, θ_min_ = 1.7°
Absorption correction: multi-scan (*SADABS*; Bruker, 2009)	*h* = −13→13
*T*_min_ = 0.865, *T*_max_ = 0.940	*k* = −14→8
8299 measured reflections	*l* = −15→14

### Refinement

**Table:**

Refinement on *F*^2^	Primary atom site location: structure-invariant direct methods
Least-squares matrix: full	Secondary atom site location: difference Fourier map
*R*[*F*^2^ > 2σ(*F*^2^)] = 0.043	Hydrogen site location: inferred from neighbouring sites
*wR*(*F*^2^) = 0.114	H-atom parameters constrained
*S* = 1.05	*w* = 1/[σ^2^(*F*_o_^2^) + (0.0525*P*)^2^ + 0.734*P*] where *P* = (*F*_o_^2^ + 2*F*_c_^2^)/3
5896 reflections	(Δ/σ)_max_ = 0.001
382 parameters	Δρ_max_ = 0.31 e Å^−^^3^
0 restraints	Δρ_min_ = −0.36 e Å^−^^3^

### Special details

**Table:**

Geometry. All e.s.d.'s (except the e.s.d. in the dihedral angle between two l.s. planes)are estimated using the full covariance matrix. The cell e.s.d.'s are takeninto account individually in the estimation of e.s.d.'s in distances, anglesand torsion angles; correlations between e.s.d.'s in cell parameters are onlyused when they are defined by crystal symmetry. An approximate (isotropic)treatment of cell e.s.d.'s is used for estimating e.s.d.'s involving l.s. planes.
Refinement. Refinement of *F*^2^ against ALL reflections. The weighted *R*-factor *wR* and goodness of fit *S* are based on *F*^2^, conventional *R*-factors *R* are based on *F*, with *F* set to zero for negative *F*^2^. The threshold expression of *F*^2^ > σ(*F*^2^) is used only for calculating *R*-factors(gt) *etc*. and is not relevant to the choice of reflections for refinement. *R*-factors based on *F*^2^ are statistically about twice as large as those based on *F*, and *R*- factors based on ALL data will be even larger.

### Fractional atomic coordinates and isotropic or equivalent isotropic displacement parameters (Å^2^)

**Table:**

	*x*	*y*	*z*	*U*_iso_*/*U*_eq_	
C1	0.89718 (19)	0.39500 (17)	0.31293 (18)	0.0163 (5)	
C2	0.9400 (2)	0.47051 (18)	0.21368 (19)	0.0209 (5)	
H2	0.8848	0.5138	0.1809	0.025*	
C3	1.0631 (2)	0.48184 (19)	0.16355 (19)	0.0228 (5)	
H3	1.0927	0.5342	0.0968	0.027*	
C4	1.1440 (2)	0.41738 (19)	0.20996 (19)	0.0221 (5)	
H4	1.2286	0.4261	0.1745	0.027*	
C5	1.10305 (19)	0.33986 (18)	0.30798 (18)	0.0183 (5)	
C6	1.1933 (2)	0.2638 (2)	0.3499 (2)	0.0256 (5)	
H6A	1.2054	0.2903	0.4154	0.038*	
H6B	1.2706	0.2659	0.2906	0.038*	
H6C	1.1625	0.1856	0.3708	0.038*	
C7	0.97797 (19)	0.32988 (17)	0.36211 (17)	0.0161 (4)	
C8	0.92889 (18)	0.24700 (18)	0.46638 (17)	0.0156 (4)	
C9	0.95336 (19)	0.25498 (19)	0.56798 (18)	0.0192 (5)	
C10	1.0207 (2)	0.3548 (2)	0.5828 (2)	0.0284 (6)	
H10A	0.9654	0.3989	0.6359	0.043*	
H10B	1.0519	0.4034	0.5104	0.043*	
H10C	1.0883	0.3267	0.6117	0.043*	
C11	0.9101 (2)	0.17051 (19)	0.65958 (18)	0.0216 (5)	
H11	0.9255	0.1763	0.7284	0.026*	
C12	0.8451 (2)	0.07806 (19)	0.65295 (18)	0.0210 (5)	
H12	0.8198	0.0198	0.7155	0.025*	
C13	0.81742 (19)	0.07125 (18)	0.55509 (18)	0.0181 (5)	
H13	0.7710	0.0094	0.5506	0.022*	
C14	0.85797 (18)	0.15552 (17)	0.46314 (17)	0.0141 (4)	
C15	0.84517 (18)	0.06836 (17)	0.31359 (17)	0.0141 (4)	
H15	0.8975	0.0128	0.3378	0.017*	
C16	0.80417 (18)	0.04914 (17)	0.22034 (17)	0.0135 (4)	
C17	0.84835 (18)	−0.04704 (17)	0.16927 (17)	0.0149 (4)	
H17	0.9013	−0.0975	0.1988	0.018*	
C18	0.81747 (18)	−0.07072 (17)	0.07782 (16)	0.0131 (4)	
C19	0.86198 (19)	−0.17752 (18)	0.02330 (17)	0.0161 (5)	
C20	0.9828 (2)	−0.21997 (19)	0.04188 (19)	0.0207 (5)	
H20A	1.0414	−0.1575	0.0173	0.031*	
H20B	1.0136	−0.2820	−0.0015	0.031*	
H20C	0.9706	−0.2476	0.1217	0.031*	
C21	0.7667 (2)	−0.27189 (18)	0.07538 (19)	0.0214 (5)	
H21A	0.7568	−0.2902	0.1559	0.032*	
H21B	0.7933	−0.3399	0.0399	0.032*	
H21C	0.6891	−0.2457	0.0635	0.032*	
C22	0.8794 (2)	−0.15392 (19)	−0.10353 (18)	0.0196 (5)	
H22A	0.8001	−0.1429	−0.1173	0.029*	
H22B	0.9207	−0.2186	−0.1381	0.029*	
H22C	0.9283	−0.0852	−0.1361	0.029*	
C23	0.73944 (18)	0.00725 (17)	0.03697 (17)	0.0145 (4)	
H23	0.7170	−0.0075	−0.0260	0.017*	
C24	0.69319 (18)	0.10452 (17)	0.08298 (17)	0.0135 (4)	
C25	0.61332 (19)	0.18978 (18)	0.03097 (17)	0.0167 (5)	
C26	0.6777 (2)	0.30584 (18)	−0.00721 (19)	0.0228 (5)	
H26A	0.6951	0.3312	0.0566	0.034*	
H26B	0.6252	0.3616	−0.0364	0.034*	
H26C	0.7537	0.2985	−0.0661	0.034*	
C27	0.48872 (19)	0.2012 (2)	0.11651 (19)	0.0226 (5)	
H27A	0.4992	0.2309	0.1812	0.034*	
H27B	0.4498	0.1264	0.1413	0.034*	
H27C	0.4378	0.2535	0.0815	0.034*	
C28	0.5921 (2)	0.15316 (19)	−0.07287 (18)	0.0204 (5)	
H28A	0.5405	0.2093	−0.1028	0.031*	
H28B	0.5523	0.0788	−0.0522	0.031*	
H28C	0.6701	0.1480	−0.1299	0.031*	
C29	0.72460 (18)	0.12414 (17)	0.17848 (17)	0.0138 (4)	
C30	0.47315 (19)	0.1931 (2)	0.57092 (18)	0.0218 (5)	
H30	0.4501	0.2738	0.5799	0.026*	
C31	0.4867 (2)	0.1302 (3)	0.6798 (2)	0.0376 (7)	
H31A	0.5088	0.0509	0.6715	0.056*	
H31B	0.4098	0.1326	0.7389	0.056*	
H31C	0.5502	0.1661	0.6999	0.056*	
C32	0.3789 (2)	0.1398 (2)	0.5329 (2)	0.0318 (6)	
H32A	0.3795	0.1781	0.4581	0.048*	
H32B	0.2986	0.1479	0.5857	0.048*	
H32C	0.3974	0.0590	0.5301	0.048*	
C33	0.5158 (2)	0.49464 (19)	0.3183 (2)	0.0261 (5)	
H33	0.5767	0.5433	0.3318	0.031*	
C34	0.3926 (2)	0.5180 (2)	0.3958 (3)	0.0422 (7)	
H34A	0.3318	0.4700	0.3846	0.063*	
H34B	0.3715	0.5982	0.3791	0.063*	
H34C	0.3945	0.5009	0.4737	0.063*	
C35	0.5221 (3)	0.5202 (2)	0.1965 (2)	0.0408 (7)	
H35A	0.6042	0.5050	0.1508	0.061*	
H35B	0.5019	0.6004	0.1781	0.061*	
H35C	0.4643	0.4720	0.1812	0.061*	
N1	0.81686 (15)	0.15436 (14)	0.36679 (14)	0.0130 (4)	
O1	0.77580 (13)	0.38487 (12)	0.36190 (12)	0.0176 (3)	
O2	0.68068 (13)	0.21507 (12)	0.22757 (12)	0.0165 (3)	
O3	0.58760 (13)	0.19059 (12)	0.48732 (12)	0.0184 (3)	
O4	0.54740 (13)	0.37846 (12)	0.34436 (12)	0.0188 (3)	
Ti1	0.66465 (3)	0.27775 (3)	0.35782 (3)	0.01328 (12)	

### Atomic displacement parameters (Å^2^)

**Table:**

	*U*^11^	*U*^22^	*U*^33^	*U*^12^	*U*^13^	*U*^23^
C1	0.0181 (11)	0.0137 (11)	0.0200 (11)	0.0000 (8)	−0.0070 (9)	−0.0087 (9)
C2	0.0284 (13)	0.0129 (11)	0.0229 (12)	0.0010 (9)	−0.0104 (10)	−0.0019 (9)
C3	0.0301 (13)	0.0175 (12)	0.0185 (12)	−0.0056 (10)	−0.0039 (10)	0.0001 (9)
C4	0.0188 (11)	0.0226 (12)	0.0243 (12)	−0.0028 (9)	−0.0028 (9)	−0.0076 (10)
C5	0.0197 (11)	0.0165 (11)	0.0210 (12)	0.0012 (9)	−0.0072 (9)	−0.0077 (9)
C6	0.0201 (12)	0.0273 (13)	0.0312 (14)	0.0019 (10)	−0.0098 (10)	−0.0061 (11)
C7	0.0203 (11)	0.0142 (11)	0.0160 (11)	0.0001 (9)	−0.0062 (9)	−0.0068 (9)
C8	0.0144 (10)	0.0165 (11)	0.0167 (11)	0.0061 (8)	−0.0053 (8)	−0.0048 (9)
C9	0.0191 (11)	0.0210 (12)	0.0207 (12)	0.0055 (9)	−0.0088 (9)	−0.0083 (9)
C10	0.0395 (15)	0.0258 (13)	0.0271 (13)	0.0011 (11)	−0.0177 (11)	−0.0103 (11)
C11	0.0245 (12)	0.0288 (13)	0.0154 (11)	0.0076 (10)	−0.0112 (9)	−0.0063 (10)
C12	0.0247 (12)	0.0233 (12)	0.0144 (11)	0.0021 (10)	−0.0074 (9)	0.0019 (9)
C13	0.0192 (11)	0.0181 (11)	0.0171 (11)	0.0005 (9)	−0.0060 (9)	−0.0019 (9)
C14	0.0137 (10)	0.0166 (11)	0.0123 (10)	0.0063 (8)	−0.0037 (8)	−0.0052 (8)
C15	0.0126 (10)	0.0143 (11)	0.0136 (10)	0.0010 (8)	−0.0030 (8)	0.0016 (8)
C16	0.0133 (10)	0.0143 (11)	0.0121 (10)	−0.0002 (8)	−0.0026 (8)	−0.0017 (8)
C17	0.0138 (10)	0.0153 (11)	0.0147 (11)	0.0023 (8)	−0.0040 (8)	−0.0004 (8)
C18	0.0130 (10)	0.0131 (10)	0.0117 (10)	−0.0019 (8)	−0.0008 (8)	−0.0022 (8)
C19	0.0170 (11)	0.0149 (11)	0.0164 (11)	0.0018 (8)	−0.0041 (9)	−0.0042 (9)
C20	0.0205 (12)	0.0214 (12)	0.0226 (12)	0.0052 (9)	−0.0078 (9)	−0.0090 (9)
C21	0.0222 (12)	0.0166 (11)	0.0250 (12)	−0.0013 (9)	−0.0055 (10)	−0.0036 (9)
C22	0.0224 (12)	0.0197 (12)	0.0172 (11)	0.0037 (9)	−0.0056 (9)	−0.0060 (9)
C23	0.0146 (10)	0.0176 (11)	0.0116 (10)	−0.0016 (8)	−0.0044 (8)	−0.0016 (8)
C24	0.0109 (10)	0.0156 (11)	0.0128 (10)	−0.0009 (8)	−0.0022 (8)	−0.0002 (8)
C25	0.0175 (11)	0.0176 (11)	0.0163 (11)	0.0038 (9)	−0.0073 (9)	−0.0028 (9)
C26	0.0311 (13)	0.0172 (12)	0.0239 (12)	0.0025 (10)	−0.0161 (10)	0.0004 (9)
C27	0.0179 (11)	0.0302 (13)	0.0227 (12)	0.0075 (10)	−0.0089 (10)	−0.0086 (10)
C28	0.0238 (12)	0.0215 (12)	0.0186 (12)	0.0041 (9)	−0.0109 (9)	−0.0029 (9)
C29	0.0129 (10)	0.0134 (10)	0.0133 (10)	−0.0007 (8)	−0.0011 (8)	−0.0022 (8)
C30	0.0164 (11)	0.0249 (12)	0.0200 (12)	0.0023 (9)	0.0022 (9)	−0.0060 (10)
C31	0.0250 (13)	0.070 (2)	0.0160 (12)	−0.0107 (13)	−0.0032 (10)	−0.0022 (12)
C32	0.0221 (13)	0.0433 (16)	0.0285 (14)	−0.0048 (11)	−0.0090 (11)	0.0041 (12)
C33	0.0287 (13)	0.0171 (12)	0.0368 (14)	0.0058 (10)	−0.0158 (11)	−0.0067 (10)
C34	0.0365 (16)	0.0381 (16)	0.0537 (19)	0.0175 (13)	−0.0125 (14)	−0.0188 (14)
C35	0.0546 (18)	0.0239 (14)	0.0435 (17)	0.0006 (13)	−0.0211 (14)	0.0090 (12)
N1	0.0130 (9)	0.0142 (9)	0.0116 (9)	−0.0018 (7)	−0.0033 (7)	−0.0010 (7)
O1	0.0170 (8)	0.0134 (8)	0.0232 (8)	0.0016 (6)	−0.0070 (6)	−0.0032 (6)
O2	0.0189 (8)	0.0169 (8)	0.0158 (8)	0.0062 (6)	−0.0075 (6)	−0.0059 (6)
O3	0.0152 (8)	0.0217 (8)	0.0157 (8)	0.0019 (6)	−0.0017 (6)	−0.0014 (6)
O4	0.0190 (8)	0.0162 (8)	0.0227 (8)	0.0046 (6)	−0.0081 (6)	−0.0044 (6)
Ti1	0.0134 (2)	0.0140 (2)	0.0128 (2)	0.00237 (14)	−0.00424 (15)	−0.00290 (15)

### Geometric parameters (Å, º)

**Table:**

C1—O1	1.358 (3)	C21—H21C	0.9800
C1—C2	1.396 (3)	C22—H22A	0.9800
C1—C7	1.407 (3)	C22—H22B	0.9800
C2—C3	1.379 (3)	C22—H22C	0.9800
C2—H2	0.9500	C23—C24	1.391 (3)
C3—C4	1.385 (3)	C23—H23	0.9500
C3—H3	0.9500	C24—C29	1.413 (3)
C4—C5	1.395 (3)	C24—C25	1.540 (3)
C4—H4	0.9500	C25—C28	1.533 (3)
C5—C7	1.406 (3)	C25—C27	1.539 (3)
C5—C6	1.507 (3)	C25—C26	1.540 (3)
C6—H6A	0.9800	C26—H26A	0.9800
C6—H6B	0.9800	C26—H26B	0.9800
C6—H6C	0.9800	C26—H26C	0.9800
C7—C8	1.495 (3)	C27—H27A	0.9800
C8—C14	1.407 (3)	C27—H27B	0.9800
C8—C9	1.411 (3)	C27—H27C	0.9800
C9—C11	1.393 (3)	C28—H28A	0.9800
C9—C10	1.516 (3)	C28—H28B	0.9800
C10—H10A	0.9800	C28—H28C	0.9800
C10—H10B	0.9800	C29—O2	1.337 (2)
C10—H10C	0.9800	C30—O3	1.431 (2)
C11—C12	1.389 (3)	C30—C31	1.506 (3)
C11—H11	0.9500	C30—C32	1.513 (3)
C12—C13	1.380 (3)	C30—H30	1.0000
C12—H12	0.9500	C31—H31A	0.9800
C13—C14	1.390 (3)	C31—H31B	0.9800
C13—H13	0.9500	C31—H31C	0.9800
C14—N1	1.433 (3)	C32—H32A	0.9800
C15—N1	1.290 (3)	C32—H32B	0.9800
C15—C16	1.445 (3)	C32—H32C	0.9800
C15—H15	0.9500	C33—O4	1.426 (3)
C16—C17	1.404 (3)	C33—C35	1.500 (4)
C16—C29	1.408 (3)	C33—C34	1.513 (3)
C17—C18	1.377 (3)	C33—H33	1.0000
C17—H17	0.9500	C34—H34A	0.9800
C18—C23	1.408 (3)	C34—H34B	0.9800
C18—C19	1.536 (3)	C34—H34C	0.9800
C19—C20	1.532 (3)	C35—H35A	0.9800
C19—C22	1.536 (3)	C35—H35B	0.9800
C19—C21	1.539 (3)	C35—H35C	0.9800
C20—H20A	0.9800	N1—Ti1	2.2540 (17)
C20—H20B	0.9800	O1—Ti1	1.8695 (15)
C20—H20C	0.9800	O2—Ti1	1.8690 (14)
C21—H21A	0.9800	O3—Ti1	1.8005 (15)
C21—H21B	0.9800	O4—Ti1	1.7937 (14)
			
O1—C1—C2	119.10 (19)	C24—C23—H23	117.9
O1—C1—C7	120.08 (19)	C18—C23—H23	117.9
C2—C1—C7	120.8 (2)	C23—C24—C29	117.44 (18)
C3—C2—C1	119.6 (2)	C23—C24—C25	121.79 (18)
C3—C2—H2	120.2	C29—C24—C25	120.75 (18)
C1—C2—H2	120.2	C28—C25—C27	107.77 (18)
C2—C3—C4	120.4 (2)	C28—C25—C24	112.19 (17)
C2—C3—H3	119.8	C27—C25—C24	110.32 (17)
C4—C3—H3	119.8	C28—C25—C26	106.95 (17)
C3—C4—C5	120.9 (2)	C27—C25—C26	110.45 (18)
C3—C4—H4	119.5	C24—C25—C26	109.11 (17)
C5—C4—H4	119.5	C25—C26—H26A	109.5
C4—C5—C7	119.4 (2)	C25—C26—H26B	109.5
C4—C5—C6	119.2 (2)	H26A—C26—H26B	109.5
C7—C5—C6	121.3 (2)	C25—C26—H26C	109.5
C5—C6—H6A	109.5	H26A—C26—H26C	109.5
C5—C6—H6B	109.5	H26B—C26—H26C	109.5
H6A—C6—H6B	109.5	C25—C27—H27A	109.5
C5—C6—H6C	109.5	C25—C27—H27B	109.5
H6A—C6—H6C	109.5	H27A—C27—H27B	109.5
H6B—C6—H6C	109.5	C25—C27—H27C	109.5
C5—C7—C1	118.85 (19)	H27A—C27—H27C	109.5
C5—C7—C8	121.64 (19)	H27B—C27—H27C	109.5
C1—C7—C8	119.41 (18)	C25—C28—H28A	109.5
C14—C8—C9	118.26 (19)	C25—C28—H28B	109.5
C14—C8—C7	119.12 (18)	H28A—C28—H28B	109.5
C9—C8—C7	122.60 (19)	C25—C28—H28C	109.5
C11—C9—C8	119.0 (2)	H28A—C28—H28C	109.5
C11—C9—C10	118.6 (2)	H28B—C28—H28C	109.5
C8—C9—C10	122.4 (2)	O2—C29—C16	120.11 (18)
C9—C10—H10A	109.5	O2—C29—C24	120.00 (18)
C9—C10—H10B	109.5	C16—C29—C24	119.87 (18)
H10A—C10—H10B	109.5	O3—C30—C31	108.02 (18)
C9—C10—H10C	109.5	O3—C30—C32	108.65 (18)
H10A—C10—H10C	109.5	C31—C30—C32	112.3 (2)
H10B—C10—H10C	109.5	O3—C30—H30	109.3
C12—C11—C9	121.7 (2)	C31—C30—H30	109.3
C12—C11—H11	119.1	C32—C30—H30	109.3
C9—C11—H11	119.1	C30—C31—H31A	109.5
C13—C12—C11	119.7 (2)	C30—C31—H31B	109.5
C13—C12—H12	120.1	H31A—C31—H31B	109.5
C11—C12—H12	120.1	C30—C31—H31C	109.5
C12—C13—C14	119.5 (2)	H31A—C31—H31C	109.5
C12—C13—H13	120.2	H31B—C31—H31C	109.5
C14—C13—H13	120.2	C30—C32—H32A	109.5
C13—C14—C8	121.63 (19)	C30—C32—H32B	109.5
C13—C14—N1	118.78 (19)	H32A—C32—H32B	109.5
C8—C14—N1	119.33 (18)	C30—C32—H32C	109.5
N1—C15—C16	125.63 (18)	H32A—C32—H32C	109.5
N1—C15—H15	117.2	H32B—C32—H32C	109.5
C16—C15—H15	117.2	O4—C33—C35	109.34 (19)
C17—C16—C29	119.69 (19)	O4—C33—C34	109.3 (2)
C17—C16—C15	117.90 (18)	C35—C33—C34	113.3 (2)
C29—C16—C15	122.40 (18)	O4—C33—H33	108.3
C18—C17—C16	122.20 (18)	C35—C33—H33	108.3
C18—C17—H17	118.9	C34—C33—H33	108.3
C16—C17—H17	118.9	C33—C34—H34A	109.5
C17—C18—C23	116.56 (19)	C33—C34—H34B	109.5
C17—C18—C19	122.77 (18)	H34A—C34—H34B	109.5
C23—C18—C19	120.66 (18)	C33—C34—H34C	109.5
C20—C19—C18	111.47 (17)	H34A—C34—H34C	109.5
C20—C19—C22	108.10 (17)	H34B—C34—H34C	109.5
C18—C19—C22	111.13 (17)	C33—C35—H35A	109.5
C20—C19—C21	108.87 (17)	C33—C35—H35B	109.5
C18—C19—C21	108.82 (17)	H35A—C35—H35B	109.5
C22—C19—C21	108.38 (18)	C33—C35—H35C	109.5
C19—C20—H20A	109.5	H35A—C35—H35C	109.5
C19—C20—H20B	109.5	H35B—C35—H35C	109.5
H20A—C20—H20B	109.5	C15—N1—C14	118.25 (17)
C19—C20—H20C	109.5	C15—N1—Ti1	125.66 (14)
H20A—C20—H20C	109.5	C14—N1—Ti1	113.45 (12)
H20B—C20—H20C	109.5	C1—O1—Ti1	131.40 (13)
C19—C21—H21A	109.5	C29—O2—Ti1	141.98 (13)
C19—C21—H21B	109.5	C30—O3—Ti1	136.19 (13)
H21A—C21—H21B	109.5	C33—O4—Ti1	147.12 (14)
C19—C21—H21C	109.5	O4—Ti1—O3	100.22 (7)
H21A—C21—H21C	109.5	O4—Ti1—O2	97.72 (6)
H21B—C21—H21C	109.5	O3—Ti1—O2	115.76 (7)
C19—C22—H22A	109.5	O4—Ti1—O1	95.75 (7)
C19—C22—H22B	109.5	O3—Ti1—O1	117.90 (7)
H22A—C22—H22B	109.5	O2—Ti1—O1	120.82 (7)
C19—C22—H22C	109.5	O4—Ti1—N1	177.41 (7)
H22A—C22—H22C	109.5	O3—Ti1—N1	82.33 (6)
H22B—C22—H22C	109.5	O2—Ti1—N1	80.70 (6)
C24—C23—C18	124.19 (19)	O1—Ti1—N1	83.37 (6)

### Hydrogen-bond geometry (Å, º)

Cg1 is the centroid of the C16–C18, C23, C24, C29 ring.

**Table:**

*D*—H···*A*	*D*—H	H···*A*	*D*···*A*	*D*—H···*A*
C20—H20*A*···*Cg*1^i^	0.98	2.74	3.659 (3)	156

Symmetry code: (i) −*x*+2, −*y*, −*z*.

## References

[bb1] Bruker (2009). *APEX2*, *SAINT* and *SADABS* Bruker AXS Inc., Madison, Wisconsin, USA.

[bb2] Chen, L., Zhao, L., Wang, Q., Hou, G., Song, H. & Zi, G. (2013). *Inorg. Chim. Acta*, **402**, 140–155.

[bb3] Sheldrick, G. M. (2008). *Acta Cryst.* A**64**, 112–122. 10.1107/S0108767307043930 18156677

[bb4] Westrip, S. P. (2010). *J. Appl. Cryst.* **43**, 920–925.

[bb5] Zi, G. (2011). *J. Organomet. Chem.* **696**, 68–75.

