# Supplementary figures and images for: Crystal structure of (2,4-di-tert-butyl-6-{[(6,6′-dimethyl-2′-oxido-1,1′-biphenyl-2-yl)imino]methyl}phenolato-κ3 O,N,O′)bis(propan-2-olato-κO)titanium(IV)
Source: Acta Crystallogr Sect E Struct Rep Online. 2014 Aug 16;70(Pt 9):m332. doi: 10.1107/S1600536814018455 (PMC4186137; doi:10.1107/S1600536814018455)

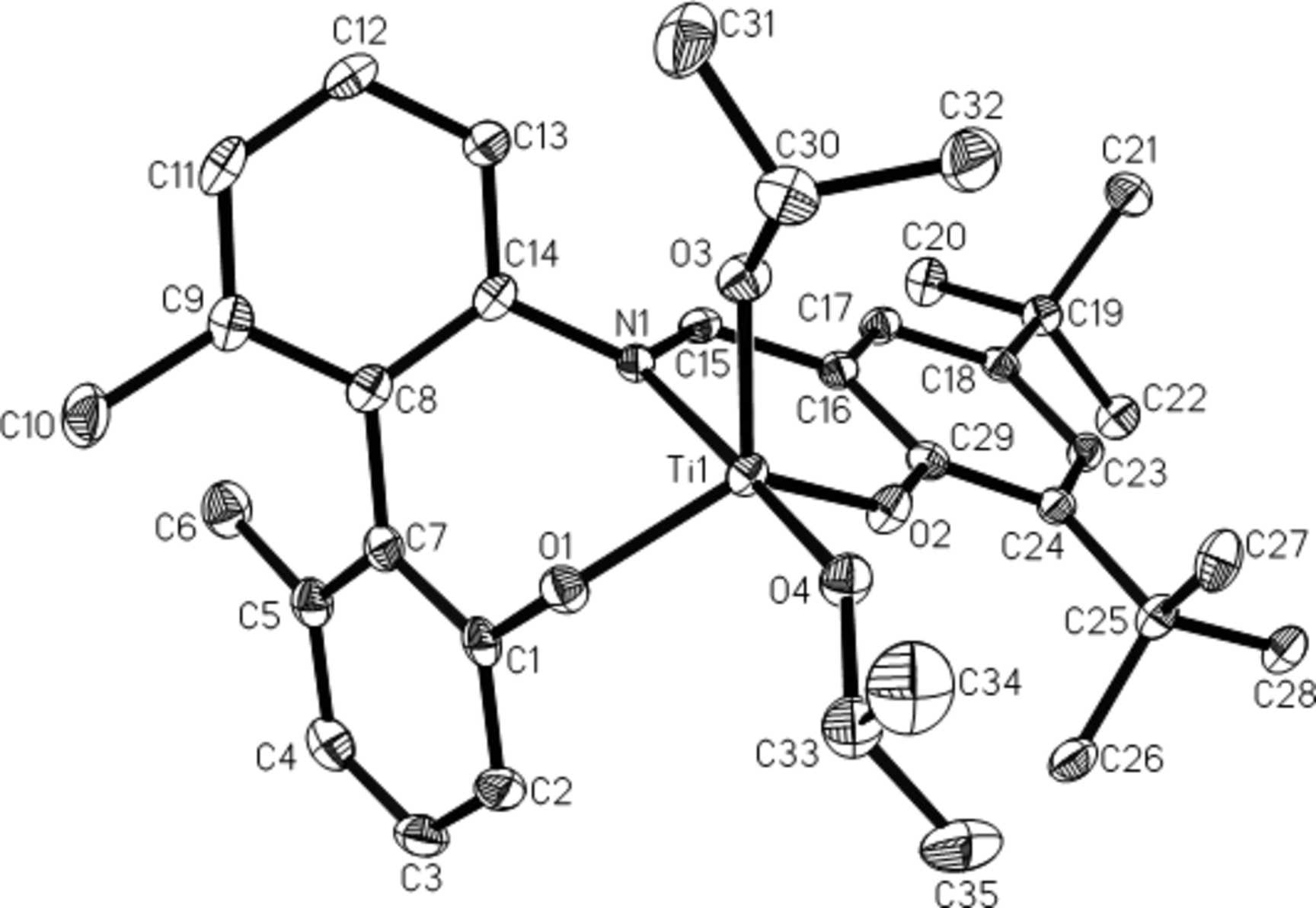

Supplement: Supplementary file 3 [file e-70-0m332-fig1.tif]
